# Preventive Effects of Dental Pulp Stem Cell-conditioned Media on Anti-RANKL Antibody-Related Osteonecrosis of the Jaw

**DOI:** 10.1007/s00223-024-01232-1

**Published:** 2024-05-29

**Authors:** Kento Kaminogo, Satoshi Yamaguchi, Hui Chen, Hideo Yagita, Naoto Toyama, Yusuke Urata, Hideharu Hibi

**Affiliations:** 1 Department of Oral and Maxillofacial Surgery, Nagoya University Graduate School of Medicine, 65 Tsurumai-cho, Showa-ku, Nagoya, Aichi 466-8550 Japan; 2 Department of Immunology, Juntendo University School of Medicine, Tokyo, Japan; 3 Department of Oral and Maxillofacial Surgery, Iwata City Hospital, Iwata, Japan; 4 Department of Oral and Maxillofacial Surgery, Nagoya University Hospital, Nagoya, Japan

**Keywords:** Medication-related osteonecrosis of the jaw, Denosumab-related osteonecrosis of the jaw, Dental pulp stem cell conditioned media, Anti-RANKL antibody

## Abstract

Medication-related osteonecrosis of the jaw is a serious disease occurring in patients with cancer and osteoporosis, who are undergoing treatment with antiresorptive agents (ARAs) such as bisphosphonate (BP) or denosumab, an antibody targeting receptor activator of NF-κB ligand. Recently, stem cell-based therapy has been shown to be effective in preventing the development of bisphosphonate-related osteonecrosis of the jaw. However, studies on denosumab-related osteonecrosis of the jaw (DRONJ) remain limited. Here, the efficacy of treatment with dental pulp stem cell conditioned media (DPSC-CM) in preventing DRONJ in a murine model was evaluated. Local administration of DPSC-CM into the extraction socket of a mouse with DRONJ decreased the number of empty osteocyte lacunae and the prevalence of ONJ. In tissues surrounding the extraction sockets in the DPSC-CM-treated group, the expression of inflammatory cytokines was attenuated and that of osteogenesis-related molecules was enhanced compared to that in the control group. Further, the expression of Wnt signaling molecules, which had been suppressed, was improved. These findings collectively suggest that DPSC-CM prevents ONJ development in a murine DRONJ model.

**Supplementary Information:**

The online version contains supplementary material available at 10.1007/s00223-024-01232-1.

## Introduction

Medication-related osteonecrosis of the Jaw (MRONJ) is a serious disease caused mainly by antiresorptive agents (ARAs). In recent years, it has been reported that other drug classes can induce osteonecrosis including the non-ARA romosozumab, the angiogenesis inhibitors bevacizumab and sunitinib, and the immunosuppressants methotrexate and everolimus. However, there are no large cohort studies supporting the causative nature of these drugs; thus, the ARA bisphosphonate (BP) and anti-receptor activator of NF-κB ligand (RANKL) antibodies are considered to be the main causes of MRONJ. Several treatment strategies have been recommended depending on the clinical stage of MRONJ, but their effectiveness is limited [1, 2].

BP-related osteonecrosis of the jaw (BRONJ) was reported in 2003 [3], and denosumab-related osteonecrosis of the jaw (DRONJ) was reported in 2010 [4]. BP and denosumab inhibit bone resorption in the same way, but their mechanisms of action differs. BP is deposited in bone and absorbed by osteoclasts, which induces apoptosis of osteoclasts themselves [5]. Denosumab is a human monoclonal antibody against RANKL, a molecule essential for osteoclast differentiation, which is secreted mainly by osteocytes, osteoblasts, and bone stromal cells. Denosumab binds to RANKL, thereby inhibiting the differentiation of osteoclast precursors into mature osteoclasts [6, 7]. Studies on DRONJ are limited because it was reported later than BRONJ. In animal studies, this limitation has also been attributed to the need for antibodies specific to the animal species [1]. This indicates the need for studies focused on denosumab as well as BP.

A combination of bone remodeling inhibition, inflammation, infection, and angiogenesis inhibition is considered to be involved in MRONJ pathogenesis, but the detailed mechanism is unknown [2]. Wnt signaling involves a family of proteins that play diverse roles in bone and cartilage formation, embryonic development, and the homeostasis of living tissues; defects in Wnt signaling are implicated in bone healing disorders, autoimmune diseases, and malignancies, thus providing therapeutic targets [8, 9]. Although previous research has demonstrated decreased expression of Wnt signaling molecules by BP administration, indicating that Wnt signaling is involved in BRONJ [10], this aspect has not been investigated in DRONJ.

In recent years, therapeutic applications with stem cells have been investigated for MRONJ [11, 12]. Although stem cells isolated from bone marrow are mostly used, they can also be isolated from dental pulp. Dental pulp can be harvested from deciduous or wisdom teeth that are extracted and discarded, such that no invasive procedure is required for harvesting. In stem cell-based therapy, the main therapeutic effect is exerted not by the cells but by factors released from the cells; thus, dental pulp stem cell conditioned medium (DPSC-CM) has attracted increasing attention as a potential therapeutic agent [13–16]. Furthermore, dental pulp stem cell-derived factors promote osteogenesis via Wnt signaling [17]. There are reports of the application of DPSC-CM in BRONJ animal models, but not DRONJ models.

As MRONJ is hard to manage once it has developed, its prevention should be targeted; to this end, this study examined the preventive effect of DPSC-CM in a murine DRONJ model, and examined its relationship with Wnt signaling.

## Materials and Methods

### DPSCs culture and DPSC-CM Preparation

DPSCs (Lonza, USA) at passage 5–8 were cultured in Dulbecco’s Modified Eagle’s Medium (DMEM) (Sigma-Aldrich, USA) supplemented with 10% FBS (Bovogen, Australia) and 1% penicillin–streptomycin solution (Fujifilm Wako, Japan) at 37 °C in an atmosphere of 5% CO_2_ until 80% confluency. Subsequently, these cells were washed with PBS (Fujifilm Wako), and the culture medium was replaced with the vehicle medium (serum-free DMEM). After a 48 h incubation, the cells were collected and centrifuged at 400×*g* and 4 °C for 3 min. The supernatants were collected and centrifuged at 1700×*g* and 4 °C for 3 min. The resulting supernatant was considered DPSC-CM and was stored at –80 °C before being used in the subsequent experiments.

### Murine DRONJ Model

**Fig. 1:**
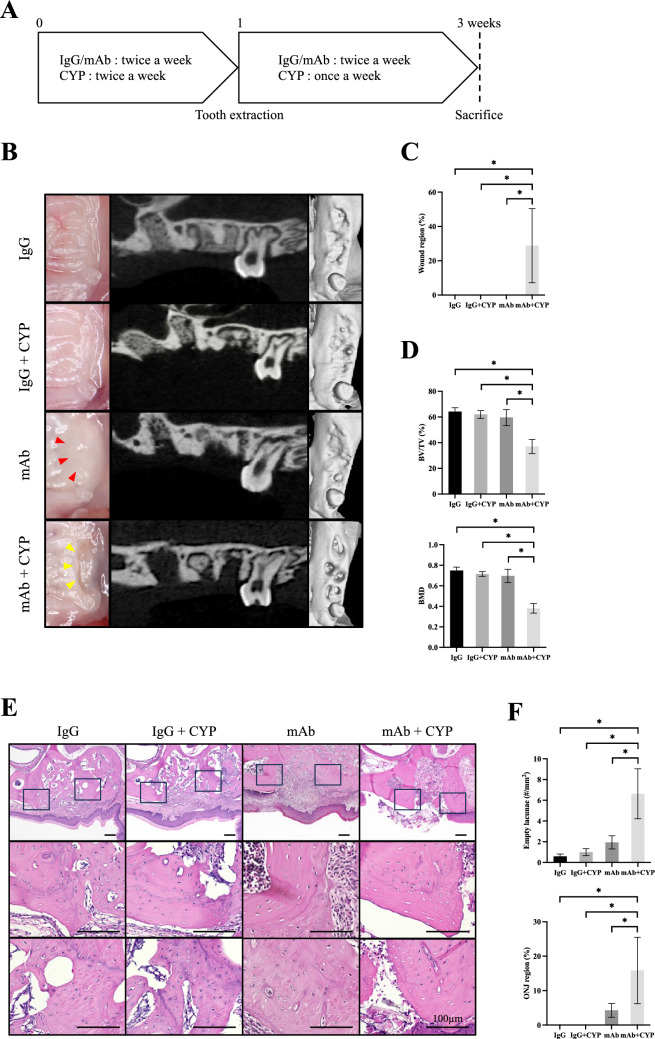
**A** Schedule for the mouse experiment. Eight-week-old male C57BL/6j mice were used in all experiments. Anti-mouse RANKL antibody (mAb, 250 µg) or normal rat IgG (250 µg) was administered intravenously, and cyclophosphamide (CYP, 150 mg/kg) was administered intraperitoneally twice a week. After 1 week, the left maxillary first and second molars were extracted. After extraction, mAb or IgG was continued twice a week and CYP once a week (n = 6 in each group). **B**–**D** The extraction wound in the mAb group was closed by the mucosa but showed edematous abnormal healing (red arrowheads). The extraction wound (yellow arrowheads) in the mAb + CYP group remained open. The maxillary bones of mice were scanned using micro-CT to calculate the bone volume/tissue volume (BV/TV) and bone mineral density (BMD) of the extraction socket. (E,F) An HE-stained histological section of the extraction socket is shown. The number of empty lacunae around the extraction socket and the ratio of necrotic maxillary bone were quantified. All data are the mean ± SD. Bar = 100 µm. *P < 0.05

Eight-week-old syngeneic male C57BL/6 J mice were purchased from Charles River Japan. The mice were housed in standard cages on a 12 h light/dark cycle and were provided water and mouse chow ad libitum. The murine DRONJ model was created according to the schedule shown in Fig. 1A. Each experimental group consisted of six animals (n = 6). The mice were administered 250 µg anti-mouse RANKL antibody (mAb) [18], 250 µg normal rat IgG (Fujifilm), or 150 mg/kg cyclophosphamide (CYP) (Shionogi, Japan) twice weekly. After one week, anesthesia (medetomidine hydrochloride and a mixture of midazolam and butorphanol tartrate) was administered intraperitoneally and the left maxillary first and second molars were extracted using a dental explorer. After extraction, mAb or IgG administration was continued twice a week, whereas CYP administration continued once a week. Three weeks after starting the experiment, the animals were euthanized by carbon dioxide inhalation. A control group was included in which mAb and CYP alone were administered. Drug doses and times of administration were based on previous studies [19, 20]. Animal that experienced abnormal bleeding or excessive tissue damage due to surgical manipulation were excluded from this study.

**Fig. 2:**
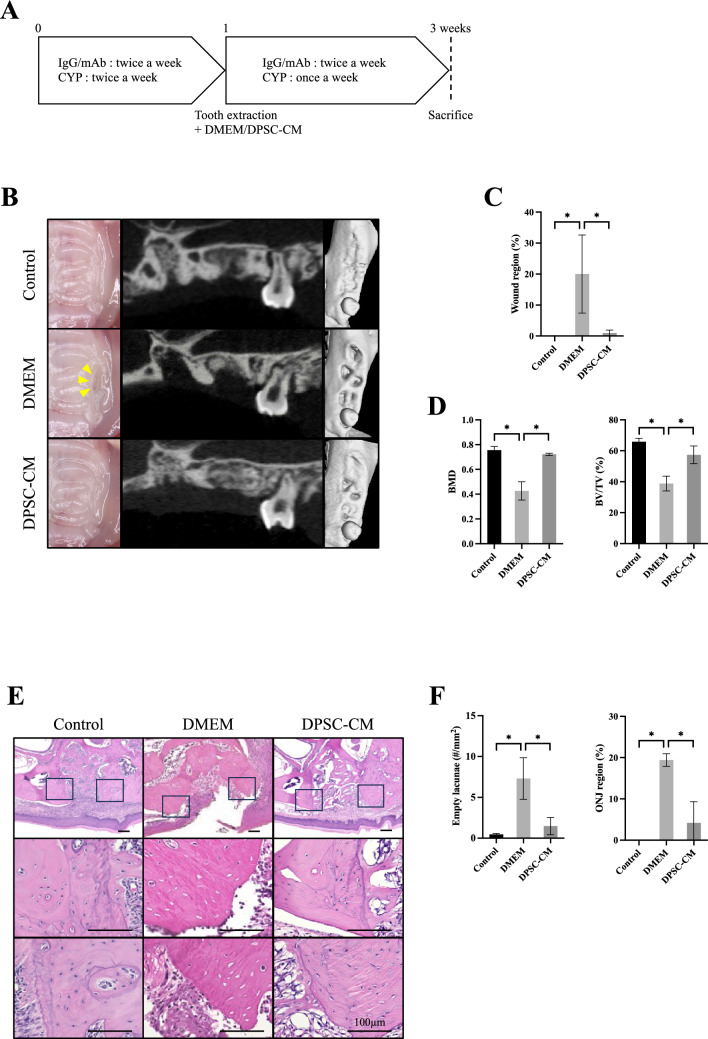
**A** Schedule of DPSC-CM or DMEM administration to mice. Twice a week, anti-mouse RANKL antibody (mAb, 250 µg) or normal rat IgG (250 µg) was administered intravenously, and cyclophosphamide (CYP, 150 mg/kg) was administered intraperitoneally. After 1 week, DPSC-CM or DMEM was administered into the extraction socket simultaneously with the extraction of the left maxillary first and second molars. After extraction, mAb or IgG was continued twice weekly and CYP once weekly (n = 6 in each group). **B**–**D** The extraction wound (yellow arrowheads) in the DMEM group remained open. The extraction wound in the DPSC-CM group was closed by the mucosa. The maxilla of the mice was scanned using micro-CT to calculate the bone volume/tissue volume (BV/TV) and bone mineral density (BMD) of the extraction socket. (E,F) An HE-stained histological section of the extraction socket is shown. The number of empty lacunae around the extraction socket and the ratio of necrotic maxillary bone were quantified. All data are the mean ± SD. Bar = 100 µm. *P < 0.05

DPSC-CM was administered to the murine DRONJ model according to the schedule in Fig. 2A. Mice treated with DMEM and untreated served as healthy controls. Atelocollagen acid solution and atelocollagen sponge MIGHTY (Koken, Japan) were used as scaffolds when DPSC-CM or DMEM was administered into the extraction socket.

### Micro-CT Scanning

The excised murine maxilla was imaged using a SkyScan1176 in vivo micro-CT scanner (Bruker, USA). The imaging conditions were set to a resolution pixel size of 9 µm and an aluminum filter of 0.5 mm; reconstruction was performed using NRecon Software (Bruker); the bone volume/tissue volume (BV/TV) and bone mineral density (BMD) of the extraction socket were quantitatively analyzed using CTAn Micro-CT Software (Bruker). The maxilla and femur were also imaged using LaTheta LCT200 (Aloka, Japan), and the respective regions of interest were constructed in 3D; trabecular bone density of the distal femur was measured using LaTheta Software (Aloka).

### Histology

The excised mouse maxilla was fixed with 4% paraformaldehyde for 48 h at 4 °C and debrided with ethanol. The tissue was then demineralized with 10% ethylenediaminetetraacetic acid (EDTA; pH 8.0) for 4 weeks. The tissue was trimmed to the appropriate size, embedded in paraffin, and sliced at a thickness of 4 µm. The sections were deparaffinized with xylene, replaced with ethanol, and stained with hematoxylin and eosin (HE). The tissue sections were photographed using an all-in-one fluorescence microscope BZ-X800 (Keyence, Japan). The ONJ region was analyzed using ImageJ software (National Institutes of Health, USA) as a region with five or more consecutive empty osteocyte lacunae [21]. An operator, unaware of the treatment condition, performed a histological evaluation.

### Immunochemistry-Paraffin (IHC-P) Protocol

The paraffin Sects. (4 µm) of mouse maxilla were immersed in 1 mM EDTA (pH 8.0) at 90 °C for 65 min for antigen activation. Endogenous peroxidase was removed using 0.3% H_2_O_2_ in methanol. Staining was then performed using a VECTASTAIN ABC-PO Kit and VECTOR DAB Substrate Kit (Vector Laboratories, USA). The primary antibodies used were anti-Wnt10b antibody (Abcam, UK), anti-beta Catenin antibody (Abcam), and anti-Dkk-1 (Proteintech Group, USA). The tissue sections were photographed using a BZ-X800 system, and the number of DAB-positive cells in the corresponding region was analyzed using machine learning with HALO imaging analysis software (Indica Labs, USA).

### Real-Time RT-PCR

Real-time RT-PCR was performed as previously reported [22]. In brief, excised tissues stored at –80 °C, were crushed in a Multi-beads Shocker (Yasui Kikai, Japan). Total RNA was isolated using the TRIzol LS Reagent (Thermo Fisher Scientific, USA), and cDNA was synthesized using ReverTra Ace qPCR RT Master Mix with gDNA Remover (Toyobo, Japan). Quantitative real-time reverse transcriptase-polymerase chain reaction (RT-PCR) was performed using the THUNDERBIRD SYBR qPCR Mix (Toyobo) and AriaMx Realtime PCR System (Agilent Technologies International Japan, Japan). Quantitative data were normalized to GAPDH expression, and the relative gene expression was calculated according to the ΔΔCt method. The primer sequences are shown in Supplemental Information (Table S1).

### Statistical Analysis

All statistical analyses were performed using Prism version 10 (GraphPad Software, USA). Welch's t test was used to determine the p value. One-way analysis of variance was used for multiple comparisons, followed by Tukey's honestly significant difference test. The results are expressed as the mean ± standard deviation of at least three independent experiments. P < 0.05 was considered statistically significant.

### Study Approval

The animal studies were conducted according to the NIH Guidelines for the Care and Use of Laboratory Animals and approved by the Nagoya University School of Medicine Animal Care and Use Committee. Ethical approval was obtained from the ethics committee of Nagoya University (approval number M230163-002).

## Results

### ONJ-like Lesions Caused by Anti-RANKL Antibodies in Mice

Experiments were performed according to the time schedule shown in Fig. 1A to generate the murine DRONJ model. Figure 1B shows the extraction socket of a mouse 2 weeks after tooth extraction via sagittal and 3D images on micro-CT. The extraction sockets of the IgG and IgG + CYP groups were epithelialized; the mAb group was also epithelialized, but some edematous abnormal healing was observed (red arrowheads); the mAb + CYP group had an open extraction wound (yellow arrowheads) and had significantly more wound regions than those in the other groups (Fig. 1C). Micro-CT was used to measure the BV/TV and BMD for comparison of hard tissue formation in the extraction socket. The mAb + CYP group showed significantly lower BV/TV and BMD compared to that of the IgG, IgG + CYP, and mAb groups (Fig. 1D).

Figure 1E shows an HE-stained section of a mouse maxilla. The extraction sockets in the IgG and IgG + CYP groups were closed by epithelium and filled with woven bone; the extraction socket in the mAb group was closed by epithelium, but the epithelium was thickened and inflammatory cells had accumulated. The mAb + CYP group showed less woven bone formation in the extraction socket. Figure 1F shows the empty lacunae and ONJ regions in each group. The mAb + CYP group showed a significant difference in ONJ regions and the mAb group also showed some ONJ regions; however, there was no statistically significant difference between the IgG and IgG + CYP groups.

### Preventive Effects of DPSC-CM in the Murine DRONJ Model

DMEM or DPSC-CM was administered into the extraction sockets of mice according to the schedule shown in Fig. 2A. The extraction sockets in the DMEM group were not closed by epithelium and were open (yellow arrowheads). In the DPSC-CM group, as in the control group, the extraction socket was closed by epithelium (Fig. 2B). The percentage of wound regions in the DPSC-CM group was significantly smaller than that in the DMEM group (Fig. 2C). Micro-CT analysis showed that the DPSC-CM group had more hard tissue formation in the extraction socket than that in the DMEM group, and both the BMD and BV/TV were significantly improved (Fig. 2D).

Figure 2E shows the HE staining of the maxilla from each group. In the DMEM group, the epithelium of the extraction socket was not closed, debris was present inside, and the amount of woven bone formation was low. In the DPSC-CM group, the extraction socket was closed by epithelium, and the amount of woven bone formation was greater than that in the DMEM group; the DPSC-CM group also showed improvement compared to the DMEM group with respect to the empty lacunae and ONJ regions (Fig. 2F).

### Real-Time RT-PCR of Tissues Around the Extraction Sockets

**Fig. 3:**
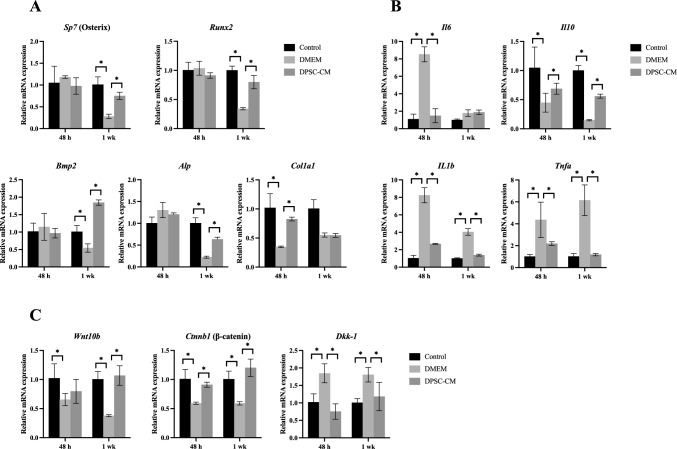
Gene expression analysis in extraction socket periapical tissues at 48 h and one week after molar extraction from healthy mice and 48 h and 1 week after DMEM and DPSC-CM treatments administered immediately after tooth extraction in the ONJ model mice (n = 6 per group). Data are the mean ± SD. *P < 0.05. **A** Expression of the osteogenesis-related genes *Sp7* (Osterix), *Runx2*, *Bmp2*, *Alp*, and *Col1a1*. **B** Expression of the inflammatory cytokine genes *Il6*, *Il10*, *Il1b*, and *Tnfa*. **C** Expression of the Wnt signaling genes *Wnt10b*, *Ctnnb1* (β-catenin), and *Dkk1*

Real-time RT-PCR was performed to examine the gene expression changes involved in osteogenesis, inflammation, and Wnt signaling in tissues around the extraction sockets. Figure 3A shows the gene expression results for osteogenesis-related molecules. The expression of Osterix (*Sp7*), runt-related transcription factor 2 (*Runx2*), bone morphogenetic protein 2 (*Bmp2*), and alkaline phosphatase (*Alp*) was significantly attenuated in the DMEM group compared to the control group one week after tooth extraction, but was enhanced in the DPSC-CM group compared to the DMEM group. The expression of Osterix, *Runx2*, *Bmp2*, and *Alp* 48 h after tooth extraction was not significantly different in any of the group. The expression of type I collagen (*Col1a1*) was significantly attenuated in the DMEM group compared to the control group at 48 h after tooth extraction and was enhanced in the DPSC-CM group compared to the DMEM group. One week after tooth extraction, *Col1a1* expression was not significantly different in each group (Fig. 3A).

Figure 3B shows the gene expression results for inflammation-related molecules. Forty-eight hours after tooth extraction, the expression of interleukin-6 (*Il6*), interleukin-1 β (*Il1b*), and tumor necrosis factor-α (*Tnfa*) was significantly enhanced in the DMEM group compared to the control and DPSC-CM groups, while interleukin-10 (*Il10*) expression was attenuated in the DMEM group compared to the control and DPSC-CM groups. One week after tooth extraction, the expression of *Il1b* and *Tnfa* was significantly enhanced in the DMEM group compared to the control and DPSC-CM groups, while the expression of *Il10* was attenuated in the DMEM group compared to the control and DPSC-CM groups.

Figure 3C shows the gene expression results of Wnt signaling-related molecules. *Wnt10b* expression 48 h after tooth extraction was attenuated in the DMEM group compared to the control group and was enhanced in the DPSC-CM group compared to the DMEM group one week after tooth extraction. Forty-eight hours and 1 week after tooth extraction, β-catenin (*Ctnnb1*) expression was more depleted in the DMEM group than in the control group, and more enhanced in the DPSC-CM group than in the DMEM group. Furthermore, 48 h and 1 week after tooth extraction, the DMEM group showed enhanced expression of dickkopf-1 (*Dkk1*) compared to that in the control group, while the DPSC-CM group showed attenuated expression compared to that in the DMEM group.

### Changes in Wnt Signaling Molecules in Murine Maxilla

**Fig. 4:**
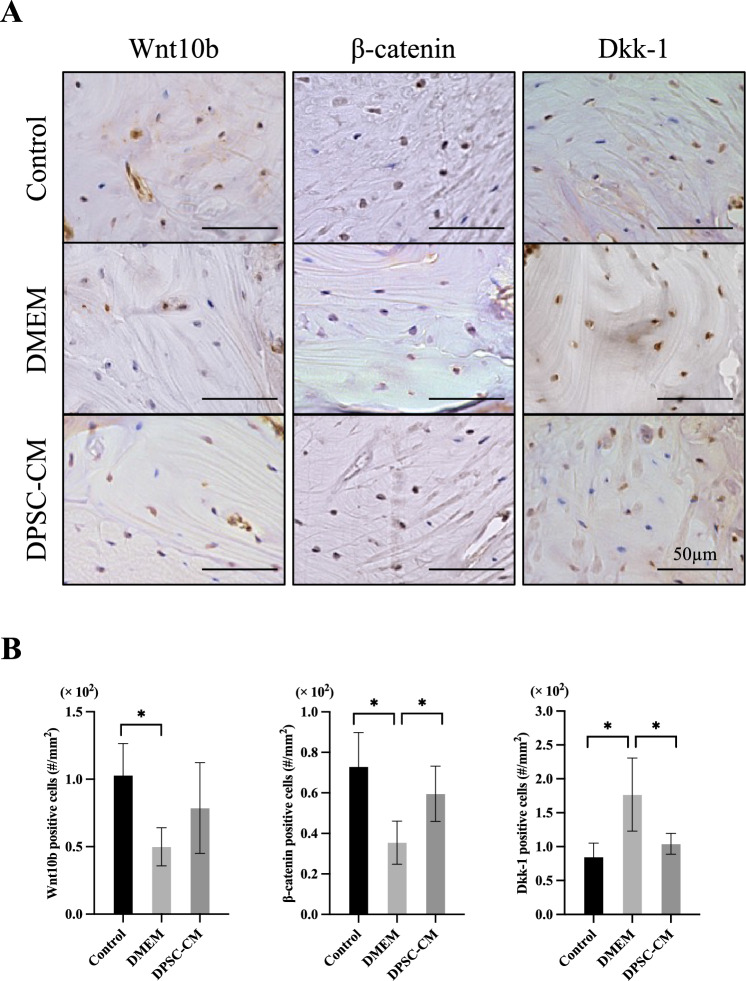
**A** Immunohistochemical staining of the extraction socket area bone two weeks after extraction of molars from healthy mice and 2 weeks after treatment with DMEM and DPSC-CM, administered immediately after the extraction of molars in the ONJ model mice (n = 6 in each group). Bar = 50 µm. **B** Number of Wnt signaling (Wnt10b, β-catenin, and Dkk-1)-positive cells (#/mm^2^) per unit area. Data are the mean ± SD. *P < 0.05

Immunohistochemical staining was performed to examine the changes in Wnt signaling in the murine maxilla. Figure 4A shows immunohistochemical staining in the tissues around the extraction socket 2 weeks after tooth extraction. With regard to Wnt10b, the number of positive cells in the region of interest was lower in the DMEM group than in the control group. The protein expression in the DPSC-CM group was not significantly different from that of other groups. The DMEM group had fewer β-catenin-positive cells in the region of interest than those in the control group, while the DPSC-CM group had more β-catenin-positive cells than in the DMEM group. Furthermore, the DMEM group had more Dkk-1-positive cells in the region of interest than those in the control group, while the DPSC-CM group had fewer Dkk-1-positive cells than those in the DMEM group (Fig. 4B).

## Discussion

MRONJ is a disease difficult to manage once it has developed, and a focus needs to be placed on prevention. This study shows that local administration of DPSC-CM prevents ONJ development in a murine DRONJ model.

The murine DRONJ model was designed to be clinically similar to the human disease. The medication was a combination of mAb and CYP. As the risk of MRONJ development in patients with cancer is high and problematic [2], a model that is more clinically relevant needs to be used. Therefore, the ONJ model was generated using the alkylating agent CYP, which is widely used to treat malignant tumors. Doses were determined based on previous reports [19, 20]. In the mAb alone group, there was no epithelial defect in the extraction socket, but half of the group showed abnormal healing with a thickened epithelium (Fig. 1B). Such epithelial hyperplasia has also been reported in a murine BRONJ model [23, 24]. Kuroshima et.al reported that the administration of an anti-RANKL antibody to gingival fibroblasts did not affect the proliferative capacity or apoptosis [25]. Therefore, the anti-RANKL antibody affected the epithelium indirectly through the suppression of osteoclasts rather than directly. In the mAb + CYP group, the extraction socket was not closed by the epithelium, and all mice showed ONJ areas; the CYP group showed no significant healing defects, indicating that ONJ is likely to develop because of synergy between mAb + CYP, consistent with clinical reports. The experiment was thus conducted using the mAb + CYP group, which produces ONJ with high probability, as a model of ONJ.

Local administration of DPSC-CM in the extraction socket of a murine DRONJ model prevents ONJ development. The scaffold used to administer DPSC-CM into the extraction socket was atelocollagen. DPSC-CM was gelatinized in an atelocollagen sponge and localized into the extraction socket. The sponge alone does not retain the liquid DPSC-CM, which easily flows out and the gel alone was difficult to retain in the extraction socket because of its high fluidity. Therefore, by combining both, the DPSC-CM could be retained in the extraction sockets. Notably, this mixture was retained over time (Supplemental Fig. S1). DPSC-CM was administered locally rather than systemically to the mice as local administration is expected to have fewer unexpected systemic effects; moreover, DPSC-CM does not interfere with the antitumor effects of anticancer drugs [26], thus reducing the risk of unexpected side effects. Indeed, there was no significant difference in the bone mineral density of the distal femur in mice, which did not interfere with the action of ARA (Supplemental Fig. S2). Furthermore, from the viewpoint of clinical application, local administration at the same time as tooth extraction in patients using ARA is simpler than intravenous administration, and thus presents fewer barriers to treatment.

Real-time RT-PCR analysis of tissues around the extraction site showed that the expression of several osteogenesis-related genes was enhanced by DPSC-CM treatment. *Sp7* (Osterix), *Runx2*, *Bmp2*, and *Alp* were significantly enhanced at one week, whereas *Col1a1* was significantly enhanced at 48 h. The expression of *Col1a1* in socket healing differs from that in healing of femoral fractures, as it is upregulated only in the early stages [27], which may reflect a characteristic of socket healing. HE staining of extraction sockets showed less internal woven bone formation in the ONJ model, which was increased by DPSC-CM administration; consistent with the histological findings, DPSC-CM has been found to promote bone formation [16], and the same effect was observed in the mouse DRONJ model. BMP2 administration has been reported to improve ONJ in a mouse BRONJ model [28], suggesting that osteogenesis is suppressed. Furthermore, the expression of inflammatory cytokines was attenuated and that of anti-inflammatory cytokines was enhanced. DPSC-CM has been reported to exert anti-inflammatory effects [13, 14, 29, 30]; similar effects were observed in the murine DRONJ model. As excessive inflammation is observed in a murine BRONJ model [31], abnormal inflammation may still be a factor in ONJ.

Wnt signaling was found to be suppressed in the DRONJ model, and this was ameliorated by administering DPSC-CM. Although Wnt signaling is reported to be suppressed in the rat BRONJ model [32, 33], some reports have shown that Dkk-1 was not suppressed, unlike in the present findings. Dkk-1 is an inhibitor of Wnt signaling and binds to the co-receptor low-density lipoprotein receptor-related protein, which activates intracellular disheveled and degrades the signaling molecule β-catenin [34]. As TNF-α increases DKK1 secretion and inhibits mesenchymal stem cell-derived osteoblastogenesis [34], elevated expression of TNF-α in the ONJ model may have been a factor in our study. Crosstalk between BMP2 and Wnt ligands has also been reported [35], and administration of BMP2 has been shown to improve BRONJ [36]. Given that Wnt10b and BMP2 expression was improved in the DPSC-CM-treated group, the observed suppression of ONJ development may be related to such crosstalk. Further, immunostaining for Wnt signaling molecules in the tissues around the extraction socket showed no significant difference between the DMEM and DPSC-CM groups with respect to Wnt10b, consistent with the real-time RT-PCR results. These results suggest that Wnt signaling is altered in the murine DRONJ model, as has been reported for the BRONJ model, which may contribute to ONJ development.

Although the involvement of Wnt signaling in ONJ development in the murine DRONJ model was suggested in this study, a murine BRONJ model was not simultaneously generated and compared. Further, the methods used to generate MRONJ models reported in the past have not been standardized [37], and the degree of inflammation and other factors are likely to differ among the models. To elucidate the detailed mechanisms involved in the development of MRONJ, both models need to be used and compared simultaneously in future research.

Overall, this study demonstrates that local administration of DPSC-CM has a prophylactic effect in a murine DRONJ model. These results and future research may provide a new treatment strategy for MRONJ.

### Supplementary Information

Below is the link to the electronic supplementary material.

Supplementary file 1 Fig. S1. Atelocollagen prepared with PBS and administered locally into the extraction sockets of healthy mice was observed over time. Atelocollagen was maintained and the extraction socket was closed with mucosal epithelium after one week. The yellow dotted lines indicate extraction sockets and atelocollagen. Fig. S2. Femurs were collected from mice in the Control, DMEM, and DPSC-CM groups, and cancellous bone density was examined using micro-CT. Data are presented as the mean ± SD. *P < 0.05. (PDF 3397 KB)
