# Supplementary material for: Preventive Effects of Dental Pulp Stem Cell-conditioned Media on Anti-RANKL Antibody-Related Osteonecrosis of the Jaw
Source: Calcif Tissue Int. 2024 May 29;115(2):185–95. doi: 10.1007/s00223-024-01232-1 (PMC11246278; doi:10.1007/s00223-024-01232-1)
Supplement: Supplementary file 1 — Fig. S1. Atelocollagen prepared with PBS and administered locally into the extraction sockets of healthy mice was observed over time. Atelocollagen was maintained and the extraction socket was closed with mucosal epithelium after one week. The yellow dotted lines indicate extraction sockets and atelocollagen. Fig. S2. Femurs were collected from mice in the Control, DMEM, and DPSC-CM groups, and cancellous bone density was examined using micro-CT. Data are presented as the mean ± SD. *P < 0.05. (PDF 3397 KB) [file 223_2024_1232_MOESM1_ESM.pdf]

Supplemental Data

Table S1

Primers used in real-time RT-PCR for mouse gene expression

Primer	Sequence (forward 5′-3′)	Sequence (reverse 5′-3′)
Gapdh	CCTGGAGAAACCTGCCAAGT	TGAAGTCGCAGGAGACAACC
Sp7 (Osterix)	GTGTTAGTAACCTGGCCGGG	CATTGGACTTCCCCCTTCTTG
Runx2	CTGTGGTTACCGTCATGGCC	GGAGCTCGGCGGAGTAGTTC
Bmp2	TGCTTCTTAGACGGACTGCG	AGCAACACTAGAAGACAGCGG
Alp	TCGGAACAACCTGACTGACC	GGTCAATCCTGCCTCCTTCC
Col1a1	GGGGCAAGACAGTCATCGAA	GGGTGGAGGGAGTTTACACG
Il6	TAGTCCTTCCTACCCCAATTTC	TTGGTCCTTAGCCACTCCTTCC
Il10	GCTCTTACTGACTGGCATGAG	CGCAGCTCTAGGAGCATGTG
IL1b	TGTCTGAAGCAGCTATGGCAA	CAGGTCATTCTCATCACTGTCAAA
Tnfa	CCCTTTACTCTGACCCCTTTATTGT	TGTCCCAGCATCTTGTGTTTCT
Wnt10b	GGGACCTCGGGTGACAATAA	CCTCTGTCCTTTTCCAACCG
Ctnnb1 (β-catenin)	TACCTGAAGCTCAGCGCAC	CTCCATCAGGTCAGCTTGAGT
Dkk1	ATCTGTCTGGCTTGCCGAAAGC	GAGGAAAATGGCTGTGGTCAGAG

Figure S1

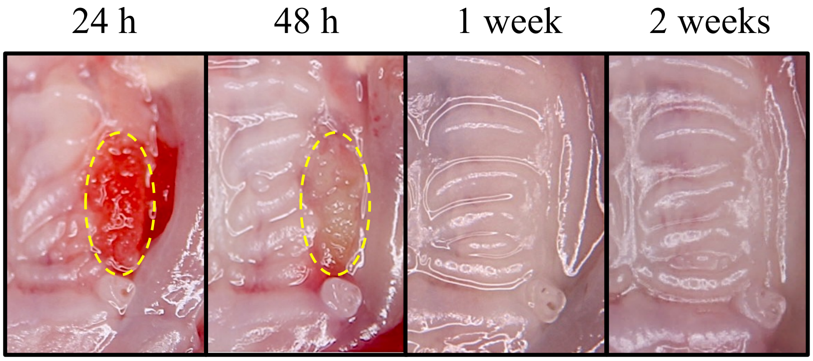

Figure S2

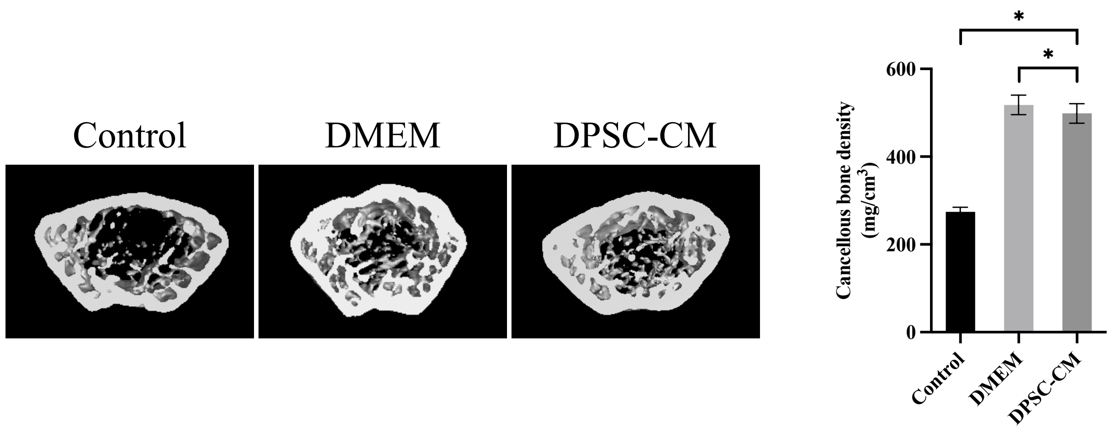
Control
DMEM
DPSC-CM
Cancellous bone density (mg/cm3)
600
400
200
0
*
*
Control
DMEM
DPSC-CM
